# Human umbilical cord-derived mesenchymal stem cells ameliorate insulin resistance by suppressing NLRP3 inflammasome-mediated inflammation in type 2 diabetes rats

**DOI:** 10.1186/s13287-017-0668-1

**Published:** 2017-11-02

**Authors:** Xiaoya Sun, Haojie Hao, Qingwang Han, Xiaoyan Song, Jiejie Liu, Liang Dong, Weidong Han, Yiming Mu

**Affiliations:** 1 Department of Endocrinology, Chinese PLA General Hospital, Beijing, 100853 China; 2 Institute of Basic Medicine Science, College of Life Science, Chinese PLA General Hospital, Beijing, 100853 China

**Keywords:** Mesenchymal stem cells, Inflammation, Insulin resistance, NLRP3 inflammasome, Palmitic acid, Lipopolysaccharide, Type 2 diabetes

## Abstract

**Background:**

Insulin resistance is one of the most common and important pathological features of type 2 diabetes (T2D). Recently, insulin resistance is increasingly considered to be associated with systemic chronic inflammation. Elevated levels of tumor necrosis factor (TNF)-α and interleukin (IL)-1β in blood are predictive indicators of the development of T2D. Mesenchymal stem cell (MSC)-based therapies have been proven to have potential immunomodulation and anti-inflammatory properties through their paracrine effects; however, the mechanism for the anti-inflammatory effect of MSCs in enhancing insulin sensitivity is still uncertain.

**Methods:**

In the present experiment, we used HepG2 cells, a human hepatoma cell line, and a MSC-HepG2 transwell culturing system to investigate the anti-inflammatory mechanism of human umbilical cord-derived MSCs (UC-MSCs) under palmitic acid (PA) and lipopolysaccharide (LPS)-induced insulin resistance in vitro. Insulin resistance was confirmed by glycogen assay kit and glucose assay kit. Inflammatory factor release was detected by ELISA, gene expression was tested by quantitative real-time PCR, and insulin signaling activation was determined by western blotting analysis. The changes of inflammatory factors and insulin signaling protein were also tested in T2D rats injected with UC-MSCs.

**Results:**

Treating HepG2 cells with PA–LPS caused NLRP3 inflammation activation, including overexpression of NLRP3 and caspase-1, and overproduction of IL-1β and IL-18 as well as TNF-α from HepG2 cells. The elevated levels of these inflammatory cytokines impaired insulin receptor action and thereby prevented downstream signaling pathways, exacerbating insulin resistance in HepG2 cells. Importantly, UC-MSCs cocultured with HepG2 could effectively alleviate PA and LPS-induced insulin resistance by blocking the NLRP3 inflammasome activation and inflammatory agents. Furthermore, knockdown of NLRP3 or IL-1β partially improved PA and LPS-induced insulin signaling impairments in the presence of UC-MSCs. Similarly, UC-MSC infusion significantly ameliorated hyperglycemia in T2D rats and decreased inflammatory activity, which resulted in improved insulin sensitivity in insulin target tissues.

**Conclusions:**

Our results indicated that UC-MSCs could attenuate insulin resistance and this regulation was correlated with their anti-inflammatory activity. Thus, MSCs might become a novel therapeutic strategy for insulin resistance and T2D in the near future.

**Electronic supplementary material:**

The online version of this article (doi:10.1186/s13287-017-0668-1) contains supplementary material, which is available to authorized users.

## Background

Pronounced changes in lifestyle and environment have made type 2 diabetes (T2D) a worldwide epidemic rapidly over the twenty-first century, and the accompanying complications constitute a main threat to global health [1]. Insulin resistance, a hallmark of T2D, is believed to be a fundamental pathologic event and underlying feature of T2D [2]. Although conventional insulin sensitizers, including metformin and rosiglitazone, have been proven to improve insulin sensitivity in target tissues, no pharmacologic agents exist which can be proven to treat diabetes completely. Hence, more efficacious strategies that can act via a new mechanism to promote insulin sensitivity are needed. Recently, insulin resistance is increasingly considered to be associated with low-grade systemic chronic inflammation, which has a central role in the pathogenesis of T2D [3, 4]. Since Hotamisligil et al. [5] first found that tumor necrosis factor (TNF)-α can be induced in a T2D rodent model, more attention has been focused on the connection between inflammation and insulin resistance. Another study in individuals revealed that, during obesity, elevated levels of C-reactive protein and interleukin (IL)-1β in the blood were predictive indicators of the development of T2D [6]. Therefore, chronic inflammation has been recognized as a critical inducer in the development of insulin resistance and T2D.

Consistent with these data, recent studies have shown that the nod-like receptor protein 3 (NLRP3) inflammasome plays a pivotal regulatory role in the mechanism that induces systemic inflammation and insulin resistance in obesity and T2D [7, 8]. The NLRP3 inflammasome can be triggered by both pathogen-associated molecular patterns and various danger-associated molecular patterns, including lipopolysaccharide (LPS) and saturated fatty acids, and further binds to its receptor on the cell surface, activating a proinflammatory pathway and inducing cytokine expression in various cell types [9, 10]. Structurally, a functional NLRP3 inflammasome is composed of NLRP3, apoptosis-associated speck-like protein (ASC), and caspase-1. NLRP3 interacts with ASC to activate caspase-1 and further regulates the maturation and secretion of proinflammatory cytokines IL-1β and IL-18, which are involved in the inflammation response [11, 12]. Indeed, multiple studies have demonstrated that inflammasome activation and the cleavage of inflammatory cytokines IL-1β and IL-18 induced by obesity in key metabolic tissues promote chronic inflammation and contribute to the development of T2D [7, 13–15]. Other researchers reported that the elevated cytokines such as caspase-1, IL-1β, IL-6, and TNF-α produced by activation of the inflammatory signaling pathways can contribute to glucose uptake failure and insulin sensitivity by disrupting insulin signaling [16–18]. Moreover, deficiency of protein in the NLRP3 inflammasome complex protects mice from high fat diet (HFD)-induced inflammation, alleviates insulin resistance, and promotes insulin signaling in insulin target tissues [8, 19, 20]. Notably, searching for an effective method to attenuate NLRP3 inflammasome-mediated inflammation will be a novel advance in treatment for insulin resistance and T2D.

Mesenchymal stem cells (MSCs) are multipotent stem cells with self-renewing capacities and low immunogenicity, which make them attractive for treating many diseases [21]. Interestingly, a recent paradigm shift suggests that MSCs have exhibited potential immunomodulation and anti-inflammatory properties through their paracrine effects [22]. In a renal medullary inflammation rat model, MSC transplantation could attenuate activation of the NLRP3 inflammasome and promote renal medullary function [23]. Moreover, MSCs have also been shown to downmodulate the inflammatory factors (IL-1β, TNF-α, and IL-6) through secreting prostaglandin E_2_ for therapies in osteoarticular diseases [24]. Additionally, promising results in a clinical trial have shown that MSCs reduce systemic inflammation in patients with T2D, and our previous study has confirmed that MSC administration alleviates insulin resistance in target tissues of HFD-treated T2D rats [25, 26]. Given the beneficial anti-inflammatory property of MSCs, it is imperative to test the possibility that MSCs could suppress NLRP3 inflammation activity to improve insulin resistance in a paracrine fashion.

In this study, we confirmed in vitro that the NLRP3 inflammasome was activated in an LPS and palmitic acid (PA)-induced inflammation model of insulin resistance in HepG2 cells. Human umbilical cord-derived MSCs (UC-MSCs) and their conditioned media (CM) could enhance insulin sensitivity through inhibiting the upregulation of NLRP3 inflammasome components with the elevated cleavage of IL-1β, IL-18, and TNF-α in insulin-resistant HepG2 cells. We also demonstrated that UC-MSCs repaired the glucose intolerance by suppressing inflammatory mediator release in insulin target tissues of the T2D animal model. Collectively, our studies provide evidence that MSC-mediated paracrine properties exert a protective effect on ameliorating insulin resistance through their immunomodulatory potency. This research further provides a rationale for the possible application of MSCs in the clinical treatment of insulin resistance and T2D.

## Methods

### Cells and cell culture

The UC-MSCs were freshly isolated from UCs of women after deliveries in the Chinese PLA General Hospital. Fibroblasts used for the experiment were obtained from cryopreserved cells from the dermis of healthy adults. UC-MSCs were isolated, amplified, and identified to meet the characteristics of MSCs using methods described previously [27, 28]. UC-MSCs were seeded and made adherent in serum-free Dulbecco’s modified Eagle’s medium (DMEM)/F12 medium (HyClone) supplemented with 10% fetal bovine serum (FBS; Hyclone) and 1× antibiotic/antimycotic (all from Invitrogen) overnight. When UC-MSCs were laid in passages 2 and 4, fresh media were collected after 48 h and centrifuged at 1000 rpm for 5 minutes with low temperature to obtain CM. The CM were then concentrated to 20 times through Vivaspin 20 (cutoff of 3 kDa; GE Healthcare UK Ltd, UK), and the concentrated CM were further filtered through a 0.22-μm syringe filter in a sterile environment and stored at −80 °C. The serum-free culture medium was concentrated as a negative control.

HepG2 cells (from ATCC) were cultured in DMEM (low glucose; Invitrogen) at 37 °C in a 5% CO_2_ atmosphere. Following starvation with serum-free DMEM containing 0.5% bovine serum albumin (BSA; Nanjing Sunshine Biotechnology Co. Ltd, China) for 16 h, 2 × 10^5^ HepG2 cells were pretreated with 10 μg/ml LPS (Sigma) and then 0.25 mM PA (Sigma) added that conjugated to fatty-acid free BSA (Sigma) for 24 h, and were then incubated with either MSC-CM or cocultured with 1 × 10^5^ UC-MSCs using transwell plants for another 24 h. A third group, which served as a background control for UC-MSCs, was cocultured with 1 × 10^5^ fibroblasts. Finally, cells were washed twice with polybutylene succinate (PBS; Sigma) and stimulated with 100 nM insulin (Sigma) during the last 3 h. Third-passage UC-MSCs were used for all of the coculturing experiments in this study.

### Animal experiment

Male Sprague–Dawley (SD) rats 8 weeks old were obtained from the Chinese PLA General Hospital and housed at a constant temperature (23 ± 1 °C) with a 12-h light and dark cycle and were allowed free access to water. For the HFD/STZ-induced T2D model, rats were given a HFD diet (40% fat, 41% carbohydrate, and 19% protein) for 8 weeks, followed by intraperitoneal injection of streptozotocin (STZ) (25 mg/kg; Sigma-Aldrich) as described previously [29]. Control rats were fed an 8-week regular chow diet. One week after STZ injection, we performed the intraperitoneal glucose tolerance tests (IPGTTs) and intraperitoneal insulin tolerance tests (IPITTs) to ensure the T2D model. At the same time, UC-MSC infusions (3 × 10^6^ MSCs suspended in 0.5 ml PBS) were administered to T2D rats via the tail vein. T2D rats receiving 0.5 ml PBS were the control group. To judge the effect of UC-MSCs, blood glucose, IPGTTs, IPITTs, the levels of serum insulin, and C-peptide were measured again at the appointed time.

### Glycogen content assay

The glycogen content of cells was measured by glycogen assay kit (Sigma-Aldrich). The blue compound generated by the reaction was assayed at 620 nm. The protein content of the collected HepG2 cells was quantified with the Bicinchoninic Acid (BCA) Protein Assay kit (Nanjing Bai Si Kai Co., China). Values were presented as the ratio of glycogen/protein (milligrams per grams).

### Glucose utilization assay

HepG2 cells were seeded and then treated with LPS, PA, and UC-MSCs at different times. Plates with medium containing 10% FBS were the control group. Glucose content in the media was assayed with a glucose assay kit (Sigma). Glucose-uptake content was obtained by the control group minus the experimental group. The glucose concentration was also normalized with the cellular protein concentration.

### Intraperitoneal glucose tolerance test and insulin tolerance test

The IPGTTs and IPITTs were performed on overnight fasted rats through injection with 2 g/kg glucose or 1 U insulin/kg intraperitoneally. At the specified time, the glucose concentrations were achieved via tail-vein blood samples. According to the area under the curve, we assessed the available-use ratio of insulin in SD rats to determine the extent of insulin resistance.

### Enzyme-linked immunosorbent assay

The concentrations of IL-1β, IL-18, and TNF-α, and serum insulin and serum C-peptide levels, were determined with specific ELISA kits (R&D Systems, Minneapolis, MN, USA).

### Quantitative real-time PCR

We measured the gene expression of inflammatory factors (NLRP3, IL-1β, IL-18, and TNF-α) with quantitative real-time PCR (qRT-PCR). Trizol reagent (Invitrogen) was used to isolate the RNA and further synthesize single strands of cDNA using a mix of oligo(dT) and random primers with the Superscript RT Kit (all from Thermo Fisher Scientific, Waltham, MA, USA) according to the manufacturer’s instructions. By following the appropriate amplification conditions for each set of primers, NLRP3, IL-1β, IL-18, and TNF-α measurements were performed on a 7,500 Fast Real-Time PCR instrument (Applied Biosystems, Foster City, CA, USA). β-actin was used as the reference gene.

### Caspase-1 activity assay

HepG2 cells in all groups were scraped in cell lysis buffer; for all groups, reaction buffer and YVAD-AFC substrate were further added to measure the activation of caspase-1 according to the instructions of a commercially available caspase-1 activity assay kit (Abcam, Cambridge, UK).

### Western blot analysis

Sample protein concentrations of cells or tissues were determined by BCA Protein Assay kit. Typically, total protein (40 μg) was electrophoresed by 8% SDS-PAGE and then transferred onto PVDF membranes. Primary antibodies used in this study were anti-NLRP3, anti-IRS (for phosphor-Ser^307^IRS), anti-IRS, anti-PI3K, anti-AKT (for phosphor-Thr^308^AKT), anti-AKT, and anti-Glut4 (all from Cell Signaling Technology, Danvers, MA, USA). After blocking, membranes were immunoblotted with primary and secondary antibodies, followed by detection with an ECL system.

### Lentiviral vectors construction and shRNA transfection to HepG2 cells

For shRNA experiments, shRNA and lentivirus were constructed by our laboratory. The sequences of NLRP3 and IL-1β are presented in Additional file 1: Figure S1. GFP lentiviral vectors were produced by transfection of 293 T cells and the viral titers reached 2 × 10^9^ TU/ml for further studies. The HepG2 cells were transfected with lentiviruses for NLRP3 and IL-1β. The medium was replaced 12 h later, and these shRNA HepG2 cells were incubated with PA–LPS for another 24 h. To identify the effect of RNA interference, the NLRP3 expression in the transfected cells treated with NLRP3-shRNA lentivirus or control-shRNA lentivirus and nontransfected HepG2 cells were detected by qRT-PCR and western blotting analysis. For IL-1β-shRNA, the gene expression and protein level of IL-1β were quantified among these groups with the same methods.

### Statistical analysis

We use Student’s *t* test to determine significant differences between two groups and one-way analysis of variance (ANOVA) to compare mean values with more than two groups. Values of *P* < 0.05, *P* < 0.01, and *P* < 0.001 were considered statistically significant. All data are represented as mean ± standard deviation.

## Results

### PA–LPS-stimulated inflammatory cytokine release in insulin-resistant HepG2 cells

**Fig. 1:**
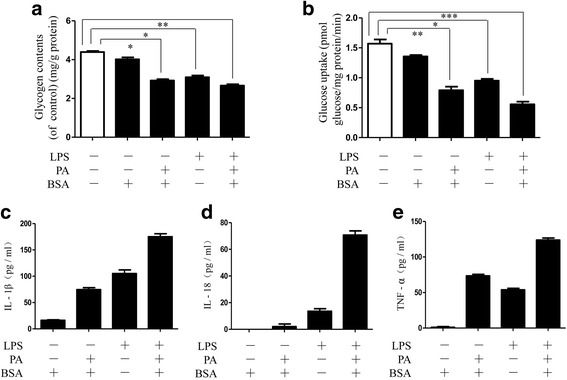
PA–LPS stimulated inflammatory cytokine release in insulin-resistant HepG2 cells. HepG2 cells were incubated with BSA, PA-loaded BSA, LPS, and PA–LPS and then stimulated with insulin (100 nm) for 3 h. (**a**) Glycogen content in cells determined using a glycogen assay kit. (**b**) A quantity of 300 μl media in all groups was measured before and after the inducer by glucose assay kit, whose difference values were further normalized with cellular protein concentration. (**c–e**) Levels of IL-1β, IL-18, and TNF-α quantified by a commercial ELISA kit. *P < 0.05, **P < 0.01, ***P < 0.001. BSA bovine serum albumin, IL interleukin, LPS lipopolysaccharide, PA palmitic acid, TNF tumor necrosis factor

PA–BSA combined with LPS treatment is known to be a common insulin-resistance inducer, so we choose PA–BSA plus LPS (PA–LPS) to stimulate HepG2 cells and induce the insulin-resistance model. PA–LPS treatment significantly decreased the glycogen content compared with the blank group, although the PA–BSA or LPS-alone group also showed partially impaired glycogen synthesis (Fig. 1a). Moreover, success of the insulin resistance model was further confirmed by an obvious reduction in insulin-stimulated glucose uptake shown in the PA–LPS group (Fig. 1b). Next, we investigated the effect of PA–LPS on the production of inflammatory cytokines. By and large, PA–LPS evoked substantial IL-1β release with low production in either the PA–BSA or LPS-alone group (Fig. 1c). The secretions of IL-18 and TNF-α were also increased the most in HepG2 cells treated with PA–LPS complex (Fig. 1d, e). These results indicate that PA–LPS could induce expression of inflammatory mediators in insulin resistance of HepG2 cells.

### UC-MSCs attenuated inflammation and improved insulin sensitivity in vitro

**Fig. 2:**
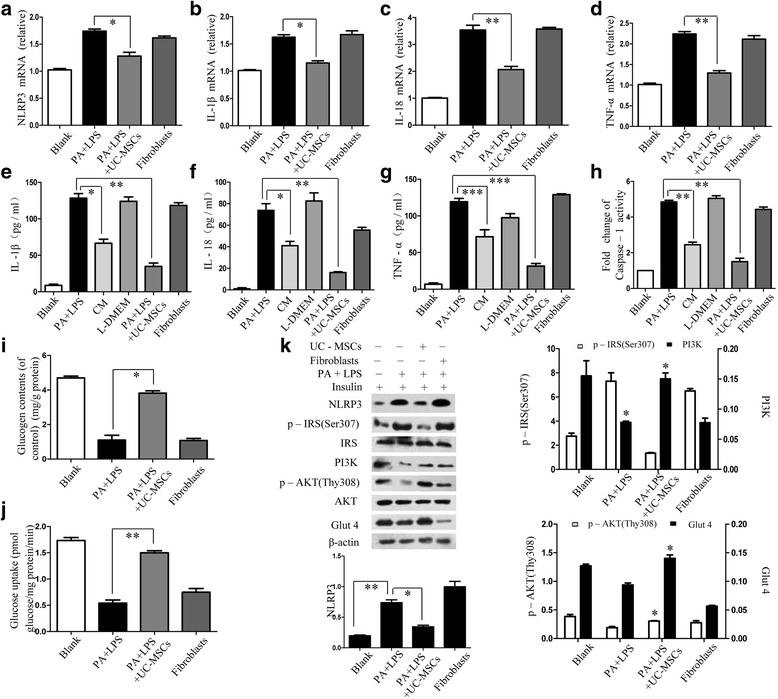
UC-MSCs attenuated PA–LPS-induced inflammation and improved insulin sensitivity in vitro. HepG2 cells were treated with PA + LPS, CM, L-DMEM, UC-MSCs, and fibroblasts accordingly. (**a–d**) mRNA of NLRP3, IL-1β, IL-18, and TNF-α analyzed by quantitative RT-PCR. Values shown as fold change to the blank control. Protein levels were detected in HepG2 cells by ELISA for IL-1β (**e**), IL-18 (**f**), and TNF-α (**g**). (**h**) Activity of caspase-1 assayed for each group. (**i, j**) Glucose metabolism of HepG2 cells in different groups by glycogen assay kit or glucose assay kit. (**k**) Western blot analysis of NLRP3, p-IRS (Ser307), IRS, PI3K, p-AKT (Thr308), AKT, and Glut4 of each group. Ratios of NLRP3, PI3K, Glut4 to β-actin, p-IRS to IRS, and p-AKT to AKT were quantitated. *P < 0.05, **P < 0.01. IL interleukin, LPS lipopolysaccharide, PA palmitic acid, TNF tumor necrosis factor, UC-MSC umbilical cord-derived mesenchymal stem cell

To investigate the anti-inflammatory effect of UC-MSCs, the PA–LPS-treated HepG2 cells were further cocultured with UC-MSCs in a transwell system. The fibroblasts were used as the control. To identify the characteristics of UC-MSCs, morphology (Additional file 2: Figure S2a), adipocytic and osteoblastic differentiation capacity (Additional file 2: Figure S2b, c), and phenotypes (Additional file 2: Figure S2d) were analyzed. Analyzing genes encoding inflammatory molecules (NLRP3, IL-1β, IL-18, and TNF-α), RNA expression revealed lower expression in the UC-MSC-treated group compared with the PA–LPS group (Fig. 2a–d). As recent studies reported that secretomes from stem cells had considerable anti-inflammatory potential [30–32], we also administered the CM into the insulin-resistant HepG2 cell and observed significant differences for the release of IL-1β, IL-18, and TNF-α either in UC-MSCs or in the CM group compared to the PA–LPS group (Fig. 2e–g). The results revealed that MSC treatment had lower inflammatory factor production than MSC-CM. Consistently, the MSC group had lower caspase-1 activity of a 1.4-fold increase, with a 2.3-fold increase in the CM group, whereas the activity of caspase-1 in PA–LPS cells was almost 4.8-fold compared to the blank group (Fig. 2 h). These results revealed that MSCs could exert an anti-inflammatory effect partially via their paracrine role.

To further observe the effect of UC-MSCs on insulin resistance, we measured both glycogen synthesis and glucose uptake which were significantly increased in PA–LPS-treated HepG2 cells (Fig. 2i, j). Additionally, levels of NLRP3 and p-Ser^307^IRS were reduced, while proteins like PI3K and phosphorylation of AKT were elevated and Glut4 translocation was also upregulated by UC-MSC treatment (Fig. 2 k). Together, these results indicate that MSCs alleviated inflammation and promoted glucose utilization by promoting an insulin signaling pathway in HepG2 cells.

### Effect of LY294002 on PI3K signaling and inflammation

**Fig. 3:**
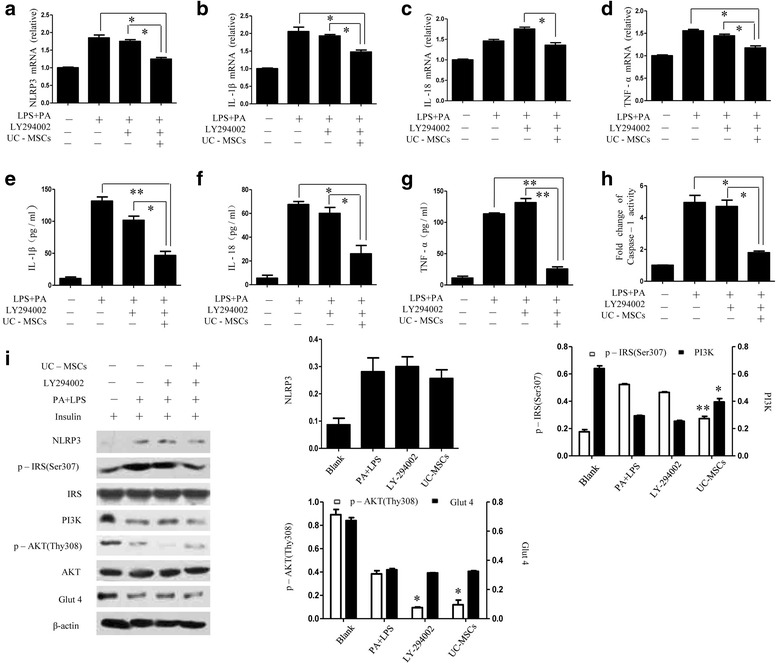
Effect of LY294002 on PI3K signaling and inflammation. (**a–d**) Quantitative RT-PCR analysis of gene expression in HepG2 cells from blank, PA + LPS, LY294002, and UC-MSC groups. (**e–g**) Levels of cytokines in cell-free culture supernatants assayed by ELISA. (**h**) Fold change of caspase-1 activity in six groups. (**i**) Immunoblotting analysis of NLRP3, p-IRS (Ser307), IRS, PI3K, p-AKT (Thr308), AKT, and Glut4 levels in HepG2 cells performed in the presence of 100 nm insulin. *P < 0.05, **P < 0.01. Images are representative of four independent experiments. IL interleukin, LPS lipopolysaccharide, PA palmitic acid, TNF tumor necrosis factor, UC-MSC umbilical cord-derived mesenchymal stem cell

To further explore the association between inflammation and the insulin signaling pathway, LY294002, a specific inhibitor of PI3K, was given 1 h before adding PA–LPS. In the pretreatment of LY294002, the gene expression of NLRP3, IL-1β, IL-18, and TNF-α was upregulated in insulin-resistant cells, which were reduced through coculturing with MSCs (Fig. 3a–d). Similarly, ELISA analysis of the supernatant showed the production of inflammatory mediators (IL-1β, IL-18, and TNF-α) was increased after PA + LPS treatment in the LY294002 group, which was significantly decreased after MSC interference (Fig. 3e–g). A similar result was obtained in caspase-1 activity assay (Fig. 3 h). Western blot analysis revealed downregulation of NLRP3 proteins by UC-MSCs, whereas decreased expression of PI3K, p-Thr^308^AKT, and Glut4 in insulin-resistant cells was not reversed by MSCs after LY294002 treatment (Fig. 3i). Hence, these data further demonstrate that UC-MSCs could attenuate inflammation without the influence of LY294002 and were able to enhance insulin sensitivity through upregulating PI3K–Akt signaling transduction.

### UC-MSCs alleviated insulin resistance in NLRP3^–/–^ and IL-1β^–/–^ HepG2 cells

**Fig. 4:**
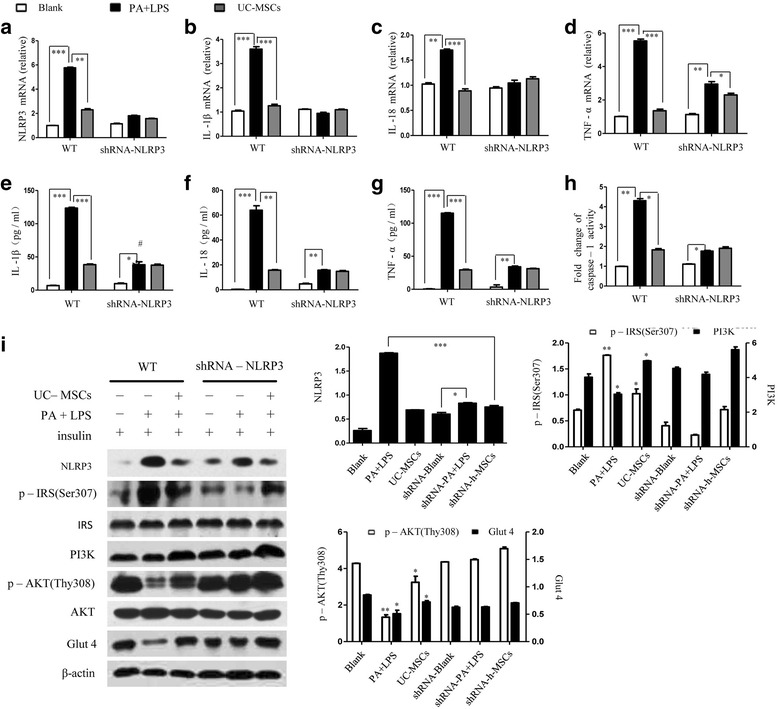
UC-MSCs alleviated insulin resistance in NLRP3^–/–^ HepG2 cells. (**a–d**) WT and shRNA-NLRP3 HepG2 cells were incubated with PA–LPS followed by coculturing with UC-MSCs and then mRNA was measured by RT-PCR. (**e–g**) Cytokine contents evaluated by ELISA assay. (**h**) Caspase-1 activity assayed in WT and shRNA-NLRP3 HepG2 cells treated with PA and UC-MSCs. (**i**) Immunoblot analysis showing the expression of NLRP3, p-IRS (Ser307), IRS, PI3K, p-AKT (Thr308), AKT, and Glut4 in three groups. *P < 0.05, **P < 0.01, ***P < 0.001. Images are representative of three independent experiments in WT and shRNA-NLRP3 HepG2 cells respectively. IL interleukin, LPS lipopolysaccharide, PA palmitic acid, TNF tumor necrosis factor, UC-MSC umbilical cord-derived mesenchymal stem cell

Next, we studied whether the NLRP3 inflammasome-dependent IL-1β production contributed to insulin resistance. HepG2 cells were transfected with shRNA for NLRP3 and IL-1β (NLRP3^–/–^ cells and IL-1β^–/–^ cells) (Additional file 3: Figure S3a, b). The results of both RT-PCR and ELISA analysis revealed that in the presence of PA–LPS, the mRNA expression and secretion of inflammatory factors were significantly lower in NLRP3^–/–^ cells compared with the WT HepG2 cells, while no significant change was seen by culturing with MSCs (Fig. 4a–g). A similar result was confirmed by caspase-1 activity (Fig. 4 h). We next examined the effect of NLRP3 on insulin receptor signaling, and protein analysis showed that NLRP3^–/–^ cells had higher insulin signaling (Fig. 4i). The results show that the insulin resistance was partially attributable to the NLRP3 inflammasome.

**Fig. 5:**
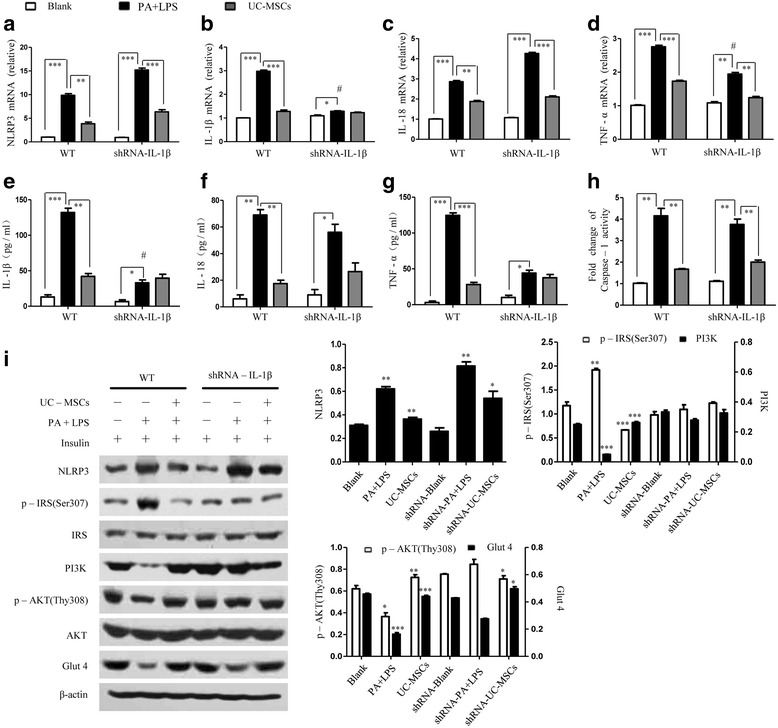
UC-MSCs alleviated insulin resistance in IL-1β^–/–^ HepG2 cells. WT and shRNA-IL-1β HepG2 cells were added to PA–LPS with or without UC-MSC preincubation. (**a–d**) Inflammatory cytokines were determined using RT-PCR. (**e–g**) Cleavage of IL-1β, IL-18, and TNF-α in media analyzed by ELISA. (**h**) Caspase-1 activity assay of WT and shRNA-IL-1β HepG2 cells in three groups. (**i**) Immunoblot analysis of relative protein levels. *P < 0.05, **P < 0.01, ***P < 0.001.﻿ # represents statistical significance between the blank and UC-MSC preincubated shRNA-IL-1β HepG2 cells. ﻿ Images are representative of three independent experiments in WT and shRNA-IL-1β HepG2 cells respectively. IL interleukin, LPS lipopolysaccharide, PA palmitic acid, TNF tumor necrosis factor, UC-MSC umbilical cord-derived mesenchymal stem cell

To better understand the role of NLRP3-dependent IL-1β, we administered PA–LPS to IL-1β^–/–^ cells and further cultured them with UC-MSCs. The transcription and secretion of IL-1β were reduced after PA–LPS stimulation. However, the levels of Nlrp3, IL-18, and TNF-α in IL-1β^–/–^ cells were increased by adding PA–LPS compared to the IL-1β^–/–^ control cells. Additionally, these effects were reversed by coculturing with UC-MSCs (Fig. 5a–g). The activity of caspase-1 was elevated after PA–LPS treatment and then reduced through culturing with UC-MSCs (Fig. 5 h). Subsequently, we assessed the effect of IL-1β on insulin signaling. Protein analysis showed lower expression of p-IRS, and elevated expression of PI3K, p-Akt, and GLUT4 in IL-1β^–/–^ cells with PA–LPS treatment (Fig. 5i). This effect was not changed by culturing with UC-MSCs. All of the data suggested that IL-1β negatively regulated insulin downstream signaling.

Taken together, these results demonstrate that NLRP3 impaired the normal insulin signaling pathway and led to insulin resistance; IL-1β, a downstream molecular of NLRP3, also played a key role in regulating insulin sensitivity. UC-MSCs could further reduce these inflammatory parameters for increasing insulin sensitivity.

### UC-MSC infusion improved hyperglycemia and insulin sensitivity in T2D rats

**Fig. 6:**
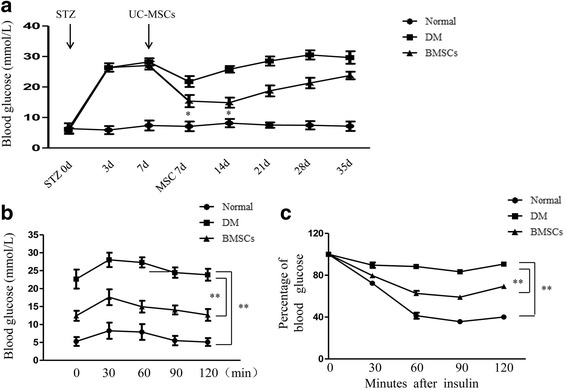
UC-MSC infusion improved hyperglycemia and insulin sensitivity in T2D rats. (**a**) Blood glucose levels determined by a glucometer ACCU-CHEK Advantage Meter in normal, diabetes mellitus (DM), and BMSC groups. (**b**) Glucose tolerance determined by the glucose concentration at different times. (**c**) Insulin tolerance assessed by IPGTT. Values are mean ± standard deviation; *n* = 8 rats each group. *P < 0.05, **P < 0.01. UC-MSC umbilical cord-derived mesenchymal stem cell

To test the hypoglycemic effect of UC-MSCs in vivo, we investigated SD rats with HFD/STZ to induce an insulin resistance model that further received UC-MSC infusion via the tail vein. The significant weight gain (data not shown) and elevation in blood glucose confirmed the success of the T2D model. From day 7 onward after UC-MSC injection, the MSC-treated rats showed an obvious decrease in blood glucose compared with a consistent hyperglycemia in untreated T2D rats, although we observed a gradual increase of blood glucose level during our study period (Fig. 6a). Moreover, the IPGTT was performed, and the results showed that UC-MSCs contributed to a significant improvement of glucose tolerance compared to the T2D group (Fig. 6b). Accordingly, insulin sensitivity was also alleviated greatly in the MSC-treated group (Fig. 6c). Moreover, serum insulin concentration declined markedly in T2D rats and MSC treatment could induce a significant increase in insulin concentration (Additional file 4: Figure S4a). The variation trend of serum C-peptide was similar to that of insulin concentration in our study (Additional file 4: Figure S4b). These results demonstrate that MSC administration contributed to amelioration of hyperglycemia and insulin resistance in T2D rats.

### UC-MSCs attenuated insulin resistance and inflammation in target tissues

**Fig. 7:**
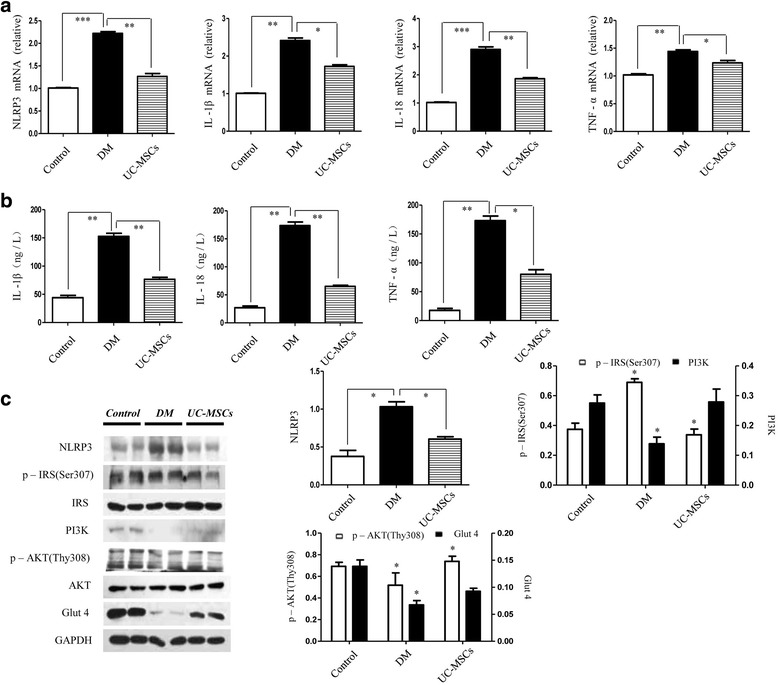
UC-MSCs attenuated HFD/STZ-induced insulin resistance and inflammation in the liver. (**a**) Gene expression of molecules involved in the inflammation of liver; results presented relative to the control WT rats. (**b**) Levels of IL-1β, IL-18, and TNF-α in the liver of all rat groups. (**c**) Immunoblotting analysis of liver in control WT rats, T2D rats, or MSC-treated rats. Relative protein levels of NLRP3, p-IRS, PI3K, p-AKT, and Glut4 are shown. *P < 0.05, **P < 0.01, ***P < 0.001. DM diabetes mellitus, IL interleukin, TNF tumor necrosis factor, UC-MSC umbilical cord-derived mesenchymal stem cell

To further address whether UC-MSCs reveal therapeutic abilities to attenuate insulin resistance in vitro, we measured the inflammatory factor production and insulin signaling transduction in liver, adipose, and skeletal muscle tissues. We particularly studied the liver because it preceded peripheral insulin resistance during T2D development. The expression of genes (NLRP3, IL-1β, IL-18, and TNF-α) in the liver was dramatically increased by HFD plus STZ; however, all genes showed restoration by MSC interference (Fig. 7a). ELISA analysis results from liver tissue also showed decreased IL-1β, IL-18, and TNF-α release after MSC treatment (Fig. 7b). Then, we continued to determine the expression of insulin signaling proteins by western blot analysis. p-Ser^307^IRS and p-Thr^308^AKT were restored greatly in the MSC-treated group, and the elevated expression of PI3K and Glut4 translocation was also observed by MSCs even with no statistical significance. Moreover, the protein analysis of NLRP3 showed decreased expression in MSC-treated rats (Fig. 7c).

**Fig. 8:**
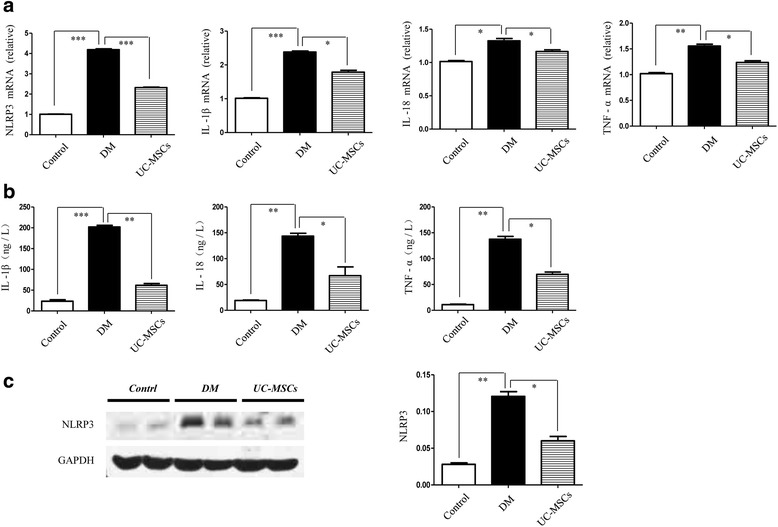
UC-MSCs attenuated HFD/STZ-induced insulin resistance and inflammation in adipose tissue. (**a**) mRNA expression of NLRP3, IL-1β, IL-18, and TNF-α of each group. (**b**) IL-1β, IL-18, and TNF-α level in the blank group, T2D group, and MSC group. (**c**) Relative protein level of NLRP3. *P < 0.05, **P < 0.01, ***P < 0.001. DM diabetes mellitus, IL interleukin, TNF tumor necrosis factor, UC-MSC umbilical cord-derived mesenchymal stem cell

Next, we continued to analyze the influence of UC-MSCs on other insulin target tissues. The mRNA expression of inflammatory cytokines (NLRP3, IL-1β, IL-18, and TNF-α) in adipose tissue was lower after MSC treatment than for T2D rats (Fig. 8a). The elevated tissue concentrations of these factors were comparable between the HFD and T2D groups, but they were also reversed by injection of MSCs (Fig. 8b). In parallel, western blot assessment revealed a decreased expression of NLRP3 in the MSC group (Fig. 8c). Similarly, as shown in muscle tissue, HFD/STZ induced a significant increase of inflammatory factors both in gene level and protein analysis; however, this inflammation action could be partially suppressed by MSC infusion (Additional file 5: Figure S5).

In summary, these in-vivo experiments indicated that UC-MSCs played an anti-inflammatory role in T2D rats and effectively improved the sensitivity of peripheral target tissues to insulin.

## Discussion

Insulin resistance is believed to be an early defect in T2D and to play a critical role through the progression from pre T2D to eventual T2D. Despite multiple drugs having been made to ameliorate insulin resistance, we still cannot ignore the limitations of accompanying side effects. Therefore, it is urgent to search for a more effective method to improve insulin resistance. Recently, attention has been focused on the link between chronic inflammation and insulin resistance [33]. The NLRP3 inflammasome and the release of inflammatory cytokines have been indicated to cause insulin resistance, and anti-inflammation therapeutic strategy is becoming promising in the treatment of T2D. Consistent with prior study, we observed inflammasome activation in an insulin resistance model of HepG2 cells, and MSC infusion not only improved insulin resistance but also impaired the activity of NLRP3 inflammation, together with a reduced caspase-1 activity and lower expression of IL-1β, IL-18, and TNF-α. Studies also showed that deletion of the genes which mediated NLRP3 and IL-1β improved glucose utilization and increased insulin sensitivity. In vivo, MSCs inhibited NLRP3 inflammasome activation and effectively promoted insulin action by stimulating the insulin receptor signaling pathway in target tissues. Hence, we concluded that treating undesired inflammation has emerged as an attractive potential therapy for insulin-resistant T2D.

MSCs have been widely explored for their multipotent differentiation capacities and nonimmunogenicity by low expression of antigen-presenting molecules [34, 35]. Some studies have further shown that systemically infused MSCs are blocked in the lung and short-lived; no viable MSCs are found in other organs [36, 37]. More interestingly, some researchers are beginning to favor the view that MSCs exert immunomodulation and anti-inflammatory effects via different paracrine mechanisms [38, 39]. Some groups had reported that mice with rheumatoid arthritis receiving MSCs were less likely to show signs of joint inflammation than those not receiving MSCs by decreasing proinflammatory cytokines such as TNF-α and IL-6 [40, 41]. Moreover, therapies in MSC-treated animal models of inflammatory bowel disease demonstrated an improved survival ratio by a reduction in T cells secreting inflammatory cytokines [42]. In our experiment, we observed that MSCs cocultured with insulin-resistant HepG2 cells could reduce inflammatory action and glucose intolerance; subsequent MSC-CM treatment further certified the anti-inflammatory effect of MSCs by their paracrine role. However, explicit analysis of factors released by MSCs that modulate immune responses and inflammatory reactions in alleviating insulin resistance still deserve to be explored.

In our study, we found that the inflammasome is coupled with the development of insulin resistance and severity of T2D. Therefore, we focused on exploring the role and mechanism of the NLRP3 inflammasome activation in insulin resistance. NLRP3 along with ASC and procaspase-1 formed the inflammasome, which mediated the mature of caspase-1 and caused the cleavage and secretion of IL-1β and IL-18 [11, 12]. The processing requires two signals: the first can be achieved by LPS stimulation to induce the IL-1β and IL-18 production from pro-IL-1β and pro-IL-18; and saturated fatty acids are the second signal to activate the inflammasome to further cause the release of procaspase-1 to caspase-1. It is well recognized that free fatty acids are substantially elevated in T2D, which become the danger signals to engage NLRP receptors and induce inflammatory factor production [43, 44], so we constructed the inflammation model by exposing HepG2 cells to LPS and then to PA. We also showed that PA–LPS treatment induced the inflammasome activation and the release of IL-1β, IL-18, and TNF-α, which could block the insulin signaling pathway. Moreover, with the lack of NLRP3 and IL-1β, our findings revealed the improved insulin signaling cascade in vitro, suggesting the NLRP3 inflammasome as an important determiner of insulin resistance. Together these data indicated that the NLRP3 inflammasome-sensing pathway contributes to inflammation in insulin resistance. However, more studies are needed both in T2D rats and humans.

Inflammasome-mediated inflammatory cytokines were recently reported to impair insulin sensitivity [19]. Here we analyze the detrimental effects of inflammation on insulin sensitive tissues (liver, adipose, muscle). As the liver is an insulin-sensitive organ and hepatic insulin resistance precedes peripheral insulin resistance [40], HepG2 cells were chosen as the insulin resistance model in vitro. It had been established that insulin binds to its receptor to trigger a series of insulin signaling transduction pathways. The IRS–PI3K–Akt pathway plays an important role in insulin’s metabolic effects. Ser-307 phosphorylation of IRS-1 is considered an important negative indicator of insulin resistance, followed by reduced PI3K/Akt phosphorylation [45]. PI3K/Akt phosphorylates and further increases the Glut4 protein content in insulin target tissues. The translocations of Glut4 to cell membranes of target tissues are responsible for improvement in sensitivity to insulin action [46]. In our study, PA–LPS-induced inflammatory factors could increase the serine phosphorylation of IRS-1, resulting in decreased phosphorylation of PI3K–Akt activation and Glut4 translocation, and MSC treatment could enhance the insulin-stimulated IRS–PI3K–Akt pathway. However, LY294002, the PI3K inhibitor, prevented p-Akt activation and eventually reduced insulin sensitivity. So, we further identified that inflammation played a role in impairing the IRS–PI3K–Akt insulin signaling pathway upon PA–LPS challenge. In vivo, we further confirmed the anti-inflammatory function of MSCs in T2D rats. NLRP3-related inflammation was observed in three key insulin-sensitive organs, and MSCs were able to decrease these inflammatory mediators and recover insulin signaling transduction. These results support the concept that the anti-inflammatory action of MSCs is virtually responsible for improving insulin resistance in target tissues of T2D rats. However, we also observed that infusion of MSCs in T2D rats was able to partially promote beta-cell function, which might be correlated with tissue repair or cytoprotective properties of MSCs. Based on these findings, one of our future directions is to research the mechanism by which MSCs ameliorate hyperglycemia in T2D and to enhance their beneficial effects.

## Conclusions

Taken together, our data indicate that stimulation of PA–LPS can impair glucose uptake, and evoke the inflammatory response characterized by overexpression of NLRP3-related proinflammatory cytokines, which further impair the IRS–PI3K–Akt signaling pathway and eventually lead to insulin resistance in HepG2 cells. MSCs effectively inhibit NLRP3 inflammasome activation and decrease these inflammatory cytokines, contributing to the amelioration of insulin resistance. In addition, MSCs also improve inflammation-related glucose intolerance in T2D rats, further certifying their beneficial role in regulating insulin sensitivity in vivo. Therefore, these results bring a new insight into the use of MSCs’ anti-inflammatory activity as another potent anti-diabetic therapy in the clinic.

## Additional files

Additional file 1: Figure S1. presenting the primer sequences of NLRP3 and IL-1β in control-shRNA cells and shRNA cells. (JPG 146 kb)

Additional file 2: Figure S2. showing characterization of UC-MSCs. (**a**) Spindle-shaped appearance observed in UC-MSC cell colonies after passage 3 under microscopy (scale bar = 500 μm). (**b, c**) Multipotential capacity of UC-MSCs showing that UC-MSCs differentiated into adipocytes with lipid vesicles in the cells (scale bar = 100 μm) and osteoblasts (scale bar = 50 μm). (**d**) Analysis of the expression of UC-MSC surface markers by flow cytometry for mesenchymal antigens. (JPG 950 kb)

Additional file 3: Figure S3. showing generation of HepG2 cell models with specific NLRP3 knockdown (NLRP3^–/–^) and IL-1β knockdown (IL-1β^–/–^). (**a**) mRNA of NLRP3 and IL-1β examined by RT-PCR. (**b**) Protein levels of NLRP3 and IL-1β assessed by immunoblotting. Data shown as mean ± standard deviation (*n* = 5). *P < 0.05. (JPG 247 kb)

Additional file 4: Figure S4. showng the variation trends of serum insulin (**a**) and serum C-peptide (**b**) in control, T2D, and MSCs rats. (JPG 128 kb)

Additional file 5: Figure S5 showing UC-MSCs attenuated HFD/STZ-induced insulin resistance and inflammation in muscle tissue. (**a**) mRNA expression of NLRP3, IL-1β, IL-18, and TNF-α of each group. (**b**) IL-1β, IL-18, and TNF-α levels in blank, T2D, and MSC groups. (**c**) Relative protein level of NLRP3 by WT. *P < 0.05, **P < 0.01, ***P < 0.001. (JPG 345 kb)

## Abbreviations

ASC: Apoptosis-associated speck-like protein
BCA: Bicinchoninic acid
BSA: Bovine serum albumin
CM: Conditioned media
DMEM: Dulbecco’s modified Eagle’s medium
FBS: Fetal bovine serum
HFD: High fat diet
IL: Interleukin
LPS: Lipopolysaccharide
MSC: Mesenchymal stem cell
NLRP3: Nod-like receptor protein 3
PA: Palmitic acid
PBS: Polybutylene succinate
SD: Sprague–Dawley
STZ: Streptozotocin
T2D: Type 2 diabetes
TNF-α: Tumor necrosis factor alpha
UC-MSC: Umbilical cord-derived MSC
