# Supplementary figures and images for: Human umbilical cord-derived mesenchymal stem cells ameliorate insulin resistance by suppressing NLRP3 inflammasome-mediated inflammation in type 2 diabetes rats
Source: Stem Cell Res Ther. 2017 Nov 2;8:241. doi: 10.1186/s13287-017-0668-1 (PMC5667486; doi:10.1186/s13287-017-0668-1)

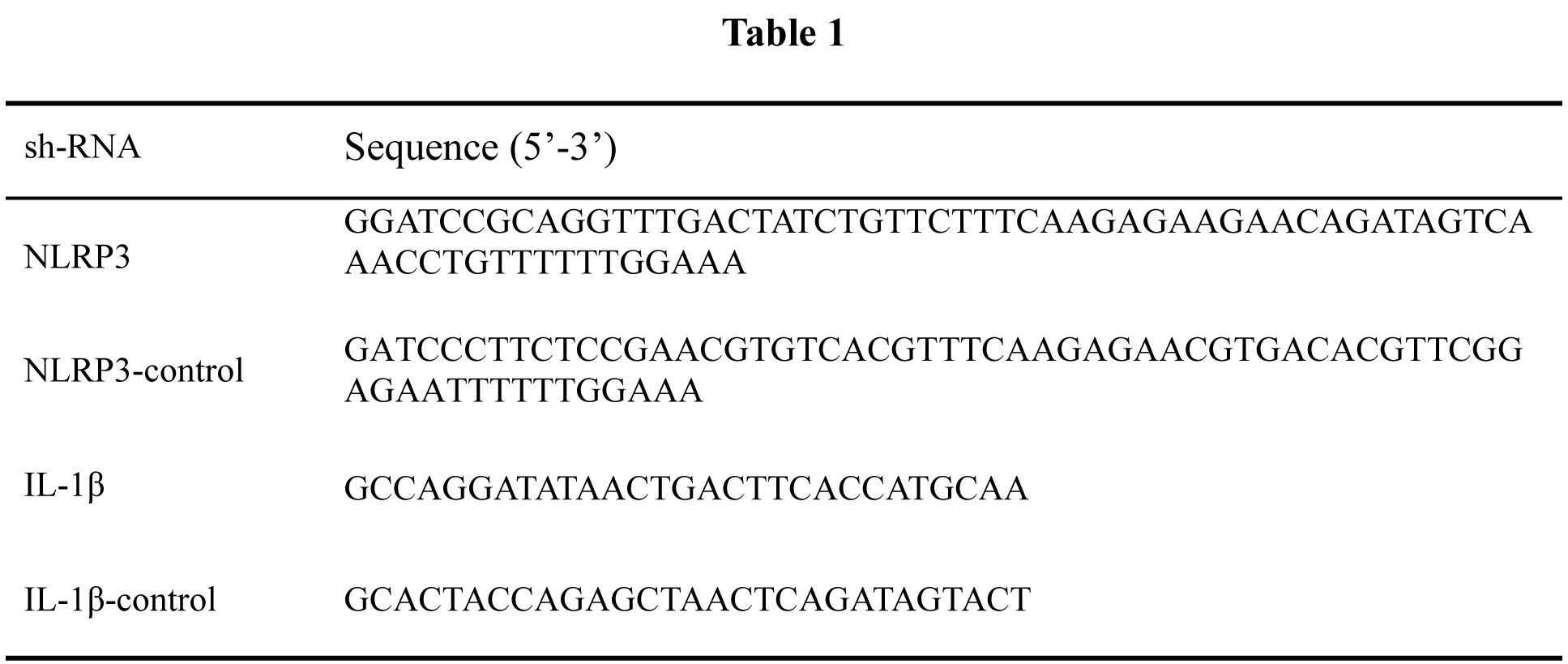

Supplement: Supplementary file 1 — presenting the primer sequences of NLRP3 and IL-1β in control-shRNA cells and shRNA cells. (JPG 146 kb) [file 13287_2017_668_MOESM1_ESM.jpg]

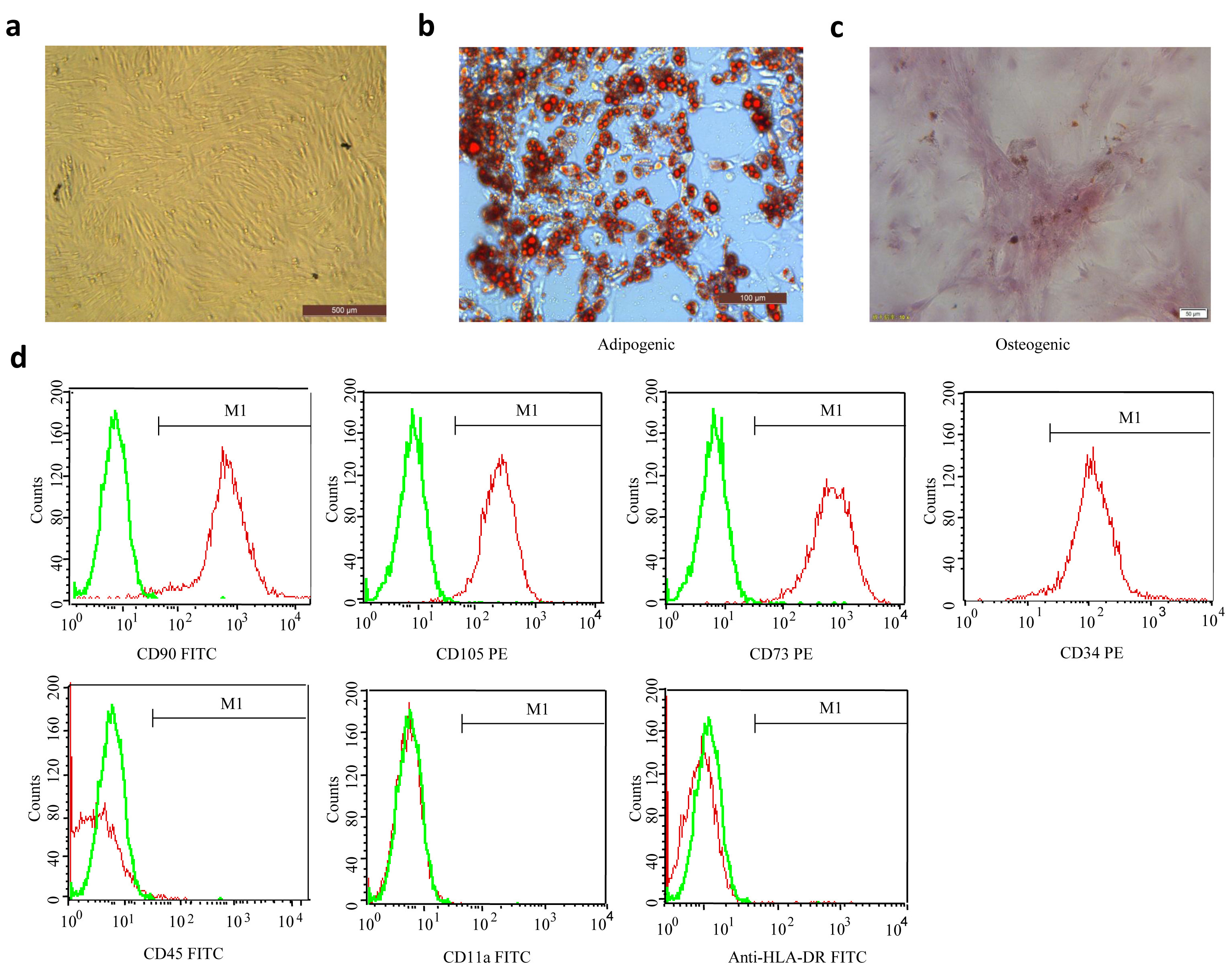

Supplement: Supplementary file 2 — showing characterization of UC-MSCs. (a) Spindle-shaped appearance observed in UC-MSC cell colonies after passage 3 under microscopy (scale bar = 500 μm). (b, c) Multipotential capacity of UC-MSCs showing that UC-MSCs differentiated into adipocytes with lipid vesicles in the cells (scale bar = 100 μm) and osteoblasts (scale bar = 50 μm). (d) Analysis of the expression of UC-MSC surface markers by flow cytometry for mesenchymal antigens. (JPG 950 kb) [file 13287_2017_668_MOESM2_ESM.jpg]

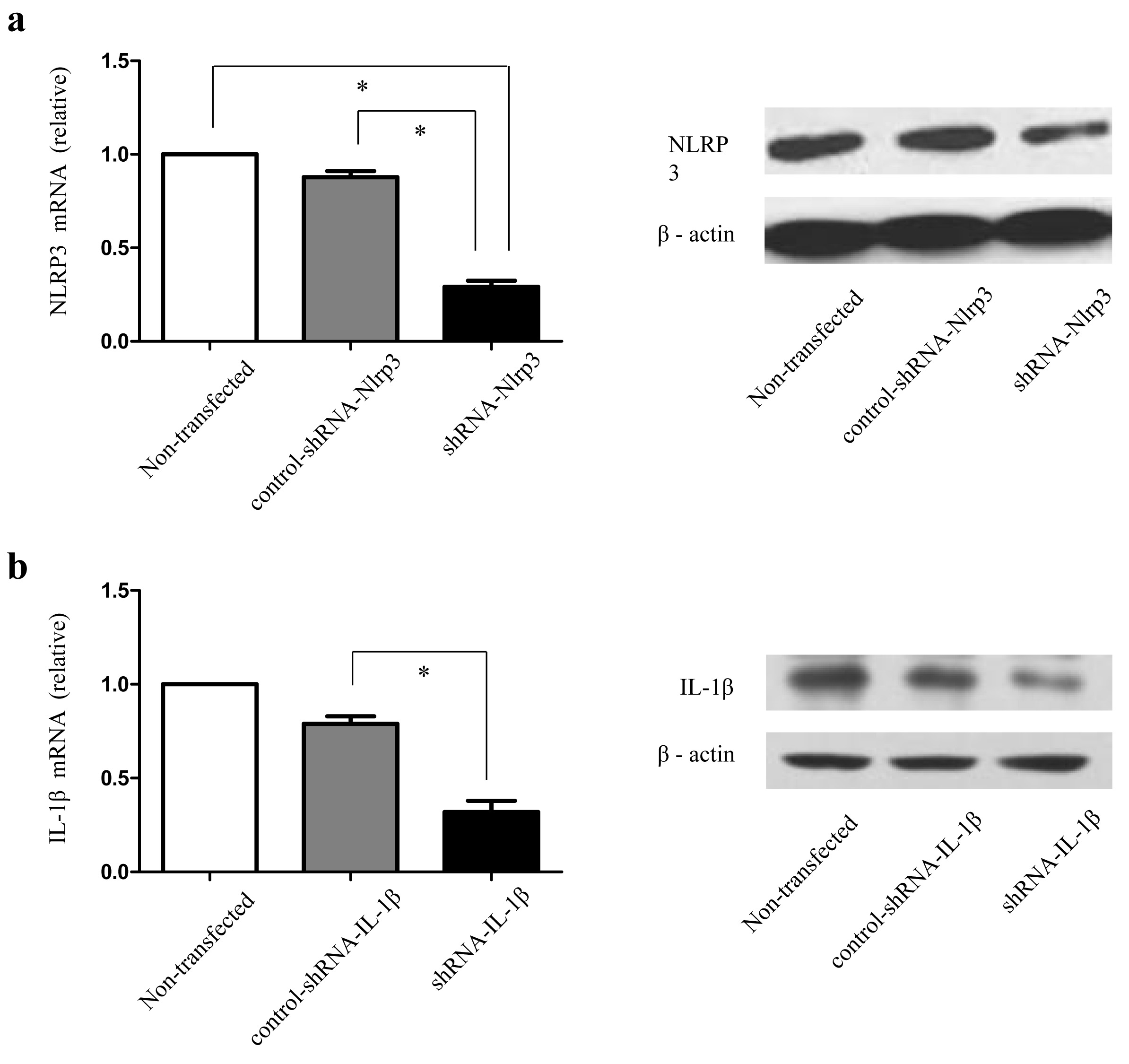

Supplement: Supplementary file 3 — showing generation of HepG2 cell models with specific NLRP3 knockdown (NLRP3–/–) and IL-1β knockdown (IL-1β–/–). (a) mRNA of NLRP3 and IL-1β examined by RT-PCR. (b) Protein levels of NLRP3 and IL-1β assessed by immunoblotting. Data shown as mean ± standard deviation (n = 5). *P < 0.05. (JPG 247 kb) [file 13287_2017_668_MOESM3_ESM.jpg]

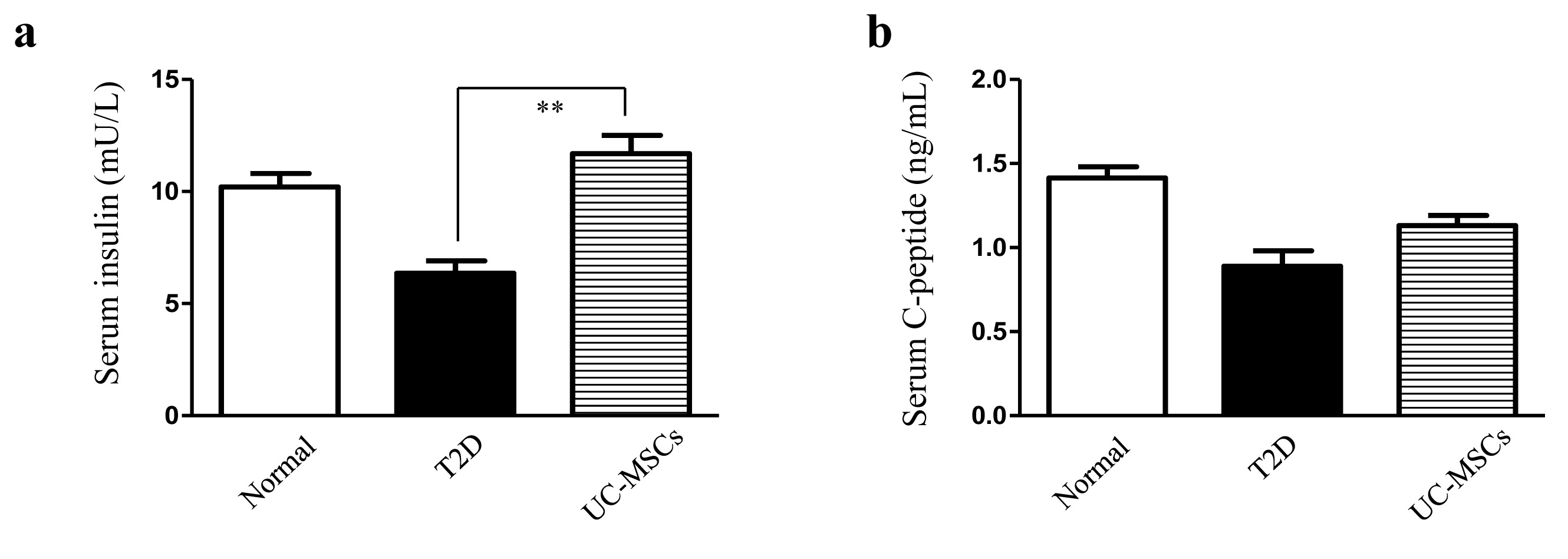

Supplement: Supplementary file 4 — showng the variation trends of serum insulin (a) and serum C-peptide (b) in control, T2D, and MSCs rats. (JPG 128 kb) [file 13287_2017_668_MOESM4_ESM.jpg]

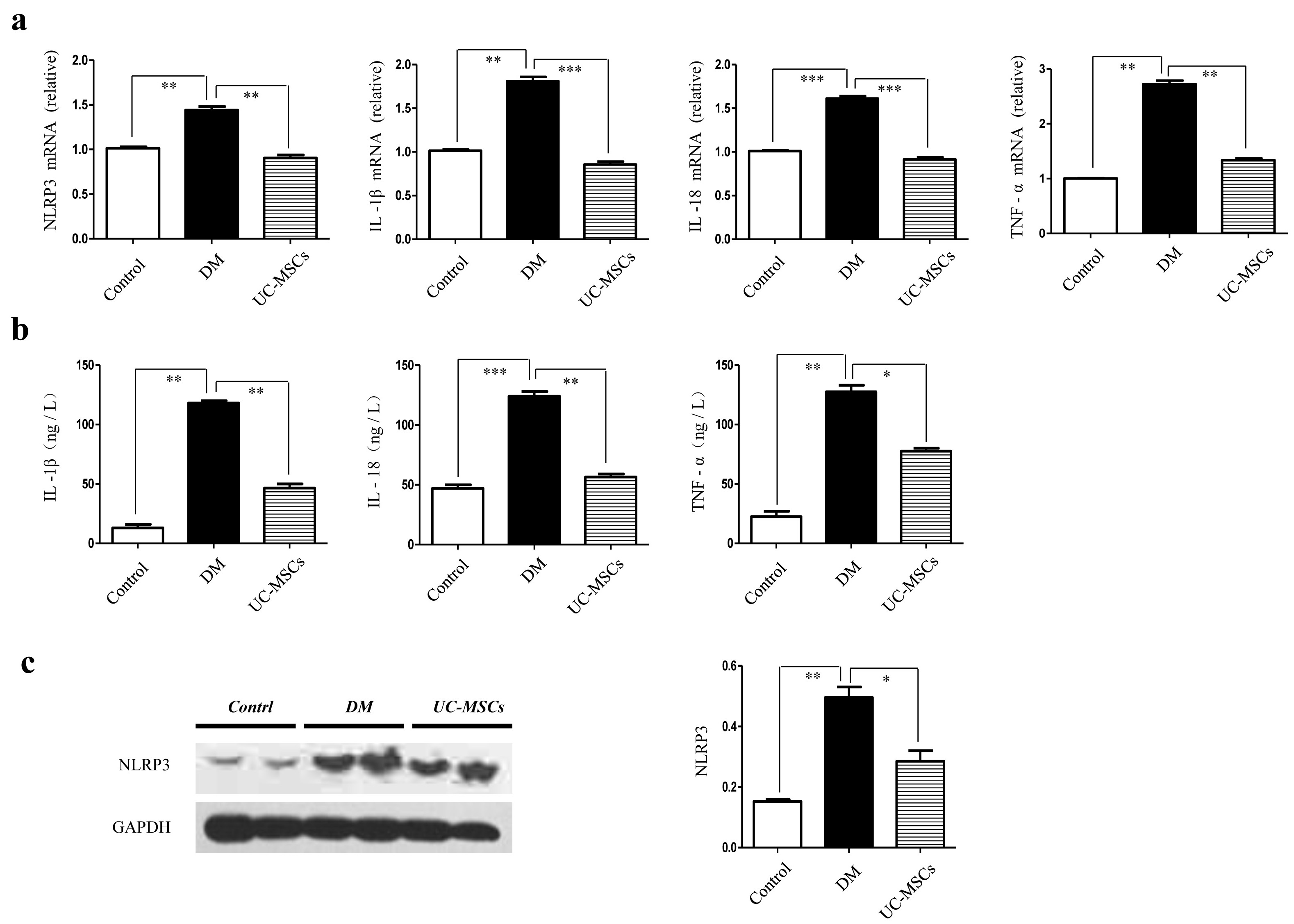

Supplement: Supplementary file 5 — showing UC-MSCs attenuated HFD/STZ-induced insulin resistance and inflammation in muscle tissue. (a) mRNA expression of NLRP3, IL-1β, IL-18, and TNF-α of each group. (b) IL-1β, IL-18, and TNF-α levels in blank, T2D, and MSC groups. (c) Relative protein level of NLRP3 by WT. *P < 0.05, **P < 0.01, ***P < 0.001. (JPG 345 kb) [file 13287_2017_668_MOESM5_ESM.jpg]
